# Modulation of Ion Channels in the Superior Cervical Ganglion Neurons by Myocardial Ischemia and Fluvastatin Treatment

**DOI:** 10.3389/fphys.2018.01157

**Published:** 2018-09-10

**Authors:** Lijun Cheng, Xinghua Wang, Tong Liu, Gary Tse, Huaying Fu, Guangping Li

**Affiliations:** ^1^Department of Cardiology, Tianjin Key Laboratory of Ionic-Molecular Function of Cardiovascular Disease, Tianjin Institute of Cardiology, The Second Hospital of Tianjin Medical University, Tianjin, China; ^2^Department of Medicine and Therapeutics, The Chinese University of Hong Kong, Shatin, Hong Kong; ^3^Li Ka Shing Institute of Health Sciences, The Chinese University of Hong Kong, Shatin, Hong Kong

**Keywords:** superior cervical ganglion, myocardial ischemia, action potential, sodium channel, delayed rectifier potassium channel, fluvastatin

## Abstract

**Background:** The superior cervical ganglion (SCG) of the autonomic nervous system plays an important role in different cardiovascular diseases. In this study, we investigated the effects of ischemia and fluvastatin treatment on the ion channel characteristics of SCG neurons in a rabbit myocardial ischemia (MI) model.

**Methods:** MI was induced by abdominal subcutaneous injections of isoproterenol (ISO). The properties of the delayed rectifier potassium channel current (*I_K_*), sodium channel current (*I_Na_*), and action potential (APs) on isolated SCG neurons in the control, MI-7d, MI-14d, fluvastatin-7d (fluvastatin pretreated 14 days and treated 7 days after ISO-induced MI), and fluvastatin-14d (fluvastatin pretreated 14 days and treated 14 days after ISO-induced MI) groups were studied. In addition, the RNA expressions of KCNQ3 and SCN9A in the SCG tissue were determined by performing real-time PCR. Intracellular calcium concentration was monitored using laser scanning confocal microscopy.

**Results:** Compared with the control group, the current amplitude of *I_K_* and *I_Na_* were increased in the MI-7d and MI-14d groups. KCNQ3 RNA (corresponding to channel proteins of *I_K_*) expression and SCN9A RNA (corresponding to channel proteins of *I_Na_*) expression were also increased in MI groups. Activation and inactivation curves for *I_Na_* in the two MI groups shifted negatively compared with the control group. These changes were reversed by fluvastatin treatment. Intracellular calcium concentration in SCG neurons was not altered significantly by MI or fluvastatin treatment. By contrast, increased AP amplitude and shortened APD_90_ were observed in the MI-7d and MI-14d groups. These changes were reversed in the fluvastatin-treated MI group.

**Conclusion:** Fluvastatin treatment partly reversed the characteristics of SCG neurons in MI. The ion channel of SCG neurons could be one of the potential targets of fluvastatin in treating coronary heart diseases.

## Introduction

The cervical ganglion (SCG) of the autonomic nervous system has been implicated in the pathogenesis of different cardiovascular diseases. Different nerve fibers arising from the SCG can innervate the heart (Hernández-Ochoa et al., 2009; He et al., 2012; Kong et al., 2013; Murakami et al., 2015). Myocardial ischemia (MI) injury is known to induce a cascade of events, which culminate in a rise of serum cardiac enzymes and in the inflammatory markers IL-6 and TNF-α, which can act on the cardiac afferent nerves. The resulting effect is increased sympathetic activity (Howard-Quijano et al., 2017). In the longer term, a large amount of norepinephrine (NE) is released from sympathetic nerves (Wu et al., 2012; Fukui et al., 2017), potentially leading to cardiac arrhythmias. Thus, both cardiac and neuronal factors could modify the outcome (Armour, 1999). Indeed, nerve block and sympathetic ganglion resection have been shown to attenuate the progression of pulmonary hypertension and suppress the remodeling of pulmonary arteries (Na et al., 2014). However, the mechanisms by which increased SCG activity aggravates MI injury have not been completely elucidated.

Statins has been widely used to protect against cerebrovascular and cardiovascular disease (Ambrosi, 2013; Schwartz et al., 2017). They can reduce the nervous injury caused by ischemia reperfusion and prevent postoperative atrial fibrillation through autonomic modulation (Liu and Li, 2006). In addition, clinical and experimental studies have suggested that statins can be used to decrease sympathetic activity (Kim et al., 2009; Lewandowski et al., 2014; Yang et al., 2016). Yet, the peripheral mechanisms involving direct actions on post-ganglionic sympathetic neurons contributing to this effect are not known.

In this study, we test the hypothesis that statins can decrease sympathetic activity by reducing electrical activity in SCG neurons to protect the myocardium. The time-dependent effects of ischemia time and of fluvastatin treatment are also explored. The characteristics of SCG neurons including ion channel properties, mRNA expression levels, intracellular calcium handling and action potential (AP) characteristics are studied.

## Materials and Methods

The experiments described in this study complied fully with the ARRIVE guidelines and were carried out in accordance with the U.K. Animals (Scientific Procedures) Act, 1986 and associated guidelines, EU Directive 2010/63/EU.

### Animal and Treatments

Young rabbits (weighing 300–500 g, and with no gender limitation) were provided by Tianjin Yuda Experimental Animal Co., Ltd. (Tianjin, China). The rabbits were randomly assigned to five groups: (a) control group (*n* = 16); (b) 7 days after isoproterenol (ISO)-induced MI (MI-7d group, *n* = 16); (c) 14 days after ISO-induced MI (MI-14d group, *n* = 16); (d) fluvastatin pretreated 14 days before and 7 days after ISO treatment (fluvastatin-7d group) (*n* = 16); and (e) fluvastatin pretreated 14 days before and 14 days after ISO treatment (fluvastatin-14d group) (animal number *n* = 16).

Myocardial ischemia was induced by abdominal subcutaneous injections of ISO (85 mg kg^-1^ d^-1^, twice at an interval of 24 h) (Qi et al., 2017) for two consecutive days. The fluvastatin group received a dose of 10 mg kg^-1^ d^-1^ for 14 days and received ISO at 85 mg kg^-1^ d^-1^ (twice at an interval of 24 h) on the 13th and 14th days. The rabbits in the control group were injected with saline. On the seventh day after the last treatment, the rabbits in the MI-7d group and the fluvastatin-7d group were sacrificed. On the 14th day after the last treatment, the rabbits in the MI-14d group and the fluvastatin-14d group were sacrificed.

### SCG Neuronal Preparations

The method used followed the same procedures as those described by Schofield and Ikeda (1988) and our previous studies (Li et al., 2010; Cheng et al., 2016). Briefly, the rabbits were anesthetized, and the SCGs were removed and placed in the incubation solution (comprising NaCl 130 mmol/L, KCl 5 mmol/L, glucose 10 mmol/L, HEPES 10 mmol/L, NaH_2_PO_4_ 1.5 mmol/L, NaHCO_3_ 25 mmol/L, MgCl_2_ 1 mmol/L, and CaCl_2_ 2 mmol/L, aerated with 95% O_2_ + 5% CO_2_ for 30 min, and adjusted to pH 7.3). Subsequently, SCG slices were cut and incubated for 30 min in the incubation solution (continuously bubbled with 95% O_2_ + 5% CO_2_). Afterward, SCG pieces were digested in another incubation solution (4 ml, adding 1.7–1.8 g/L collagenase type II (Worthington, United States), 0.6–0.7 g/L pronase E (Roche, Switzerland), and 7.0–8.0 g/L bovine serum albumin (Roche, Switzerland), 95% O_2_ + 5% CO_2_ for 50–60 min, 37°C). After digestion, SCG pieces were washed with the incubation solution and neurons were dispersed gently by glass tubes with small calibers. The neuron suspension was transferred into a culture dish for the following experiments.

### Patch-Clamp Recordings

The neuron suspension was transferred into the culture dish and attached to it for 30 min, and then washed with an external solution (comprising NaCl 130 mmol/L, KCl 5 mmol/L, MgCl_2_ 1 mmol/L, glucose 10 mmol/L, HEPES 10 mmol/L, and CaCl_2_ 2 mmol/L, aerated with 95% O_2_ + 5% CO_2_ for 30 min, and adjusted to pH 7.3) for the patch-clamp experiments.

The delayed rectifier potassium channel current (*I_K_*), sodium channel current (*I_Na_*), and AP were recorded using conventional whole-cell techniques. Electrodes had resistances in the range 2–5 MΩ. Data were recorded with an Axopatch 200B patch-clamp amplifier (Axon, United States), and stored in a computer using an interface and Clampex 10.2 (Axon, United States) data acquisition software. The tips of the glass electrodes were filled with a filtered pipette solution [comprising CsCl 134 mmol/L, NaCl 5.8 mmol/L, MgCl_2_ 1 mmol/L, HEPES 10 mmol/L, and EGTA 3 mmol/L, pH 7.2 (records of *I_Na_*); or KCl 134 mmol/L, CaCl_2_ 1 mmol/L, MgCl_2_ 2 mmol/L, HEPES 10 mmol/L, EGTA 10 mmol/L, and Na_2_ATP 2 mmol/L, pH 7.2 (records of *I_K_* and AP)]. For the measurement of *I_Na_*, 30 mmol/L TEA-Cl and 0.1 mmol/L CdCl_2_ were included in the external solution to block other channel currents. For the measurement of *I_K_*, 0.1 mmol/L CdCl_2_ was included in the external solution and neurons were held at a holding potential of -40 mV to block other channel currents. Under an inverted microscope, neurons were selected for recording. All experiments were conducted at room temperature.

### Recordings of Intracellular Calcium Concentration

The dissociated neurons were loaded for 40 min at 37°C in the incubation solution containing 3 μM Fluo-4 AM and were dissolved in dimethyl sulfoxide and Pluronic F-127 (<0.05%). The neurons were centrifuged and rinsed in the incubation solution. Fluo-4 fluorescence was monitored using a confocal laser scanning microscope (FV1000, Olympus). For fluorescence excitation, the 488-nm band of an argon laser was used. The scanning line was placed approximately equidistant between the cell membrane. The calcium peak in neurons occurs at high potassium levels (60 mM KCl). The calcium level of neurons was reported as F/F_0_, where F_0_ is the basic fluorescence value before adding KCl, and F is the peak value of fluorescence after adding KCl.

### Real-time PCR Analysis

Total RNA was extracted from the SCGs by using the RNAsimple kit (Tiangen, China) according to the manufacturer’s instructions. RNA yields and purity were determined by performing spectrophotometric analysis. Total RNA (1 μg) from each well was subjected to reverse transcription with M-MLV reverse transcriptase, dNTPs, and oligo(dT)18 primer in a total reaction volume of 20 μl. The real-time RT-PCR reaction mixture (20 μl) consisted of 8 μl double distilled water, 1 μl cDNA, 1 μl mixed primers, and 10 μl SYBR Green Mix. The thermal cycle program was run on a real-time PCR instrument (ABI 7500 Real-time PCR Detection System) according to the protocol. Experimental data were analyzed by using ABI 7500 software v2.0.6. All values obtained with the KCNQ3 (major gene corresponding to the channel protein of *I_K_*) and SCN9A (major gene corresponding to the channel protein of *I_Na_*) primers were normalized to the values obtained with the GAPDH primers. The final results were expressed as the relative integrated intensity. The primer sequences of KCNQ3 were as follows: (forward) GTACCT GCAGACGAGAATCG and (reverse) GATGGGTAGGTGAA TGCTGG. The primer sequences of SCN9A were as follows: (forward) CAACTTCTGATGACAGCGGTA and (reverse) TGTGCAAATCTGTACCACCAT.

### Statistical Analysis

All data were analyzed with Origin 6.0 for image processing and SPSS 17.0 for statistical analysis. Results were expressed as mean ± standard deviation (SD). The comparison between two groups was performed using the *t*-test and the comparison between three groups was performed using one-way ANOVA. *P* < 0.05 was considered to be statistically significant.

## Results

### Effects of MI and Fluvastatin on I - V Curves of *I_K_*

**FIGURE 1:**
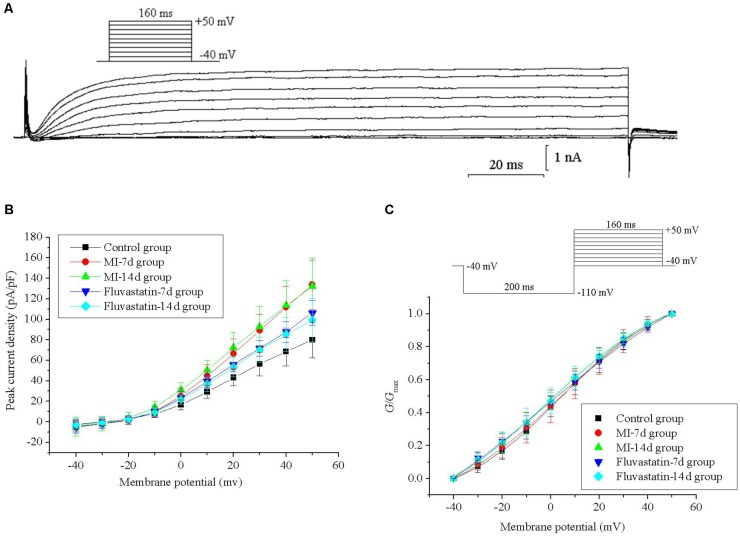
Effects of MI and fluvastatin on *I_K_* of SCG neurons. **(A)** Typical examples of *I_K_* curves. **(B)** Effects of MI and fluvastatin on I–V curves of SCG neurons *I_K_*. **(C)** Effects of MI and fluvastatin on activation curves of *I_K_*. Each point represents mean ± SD.

For constructing the I - V curves, neurons were held at a holding potential of -40 mV and stimulated by a series of 160 ms depolarizing steps from -40 to +50 mV (10 mV increment for each step). *I_K_* values were obtained and stabilized after 20–40 ms. The patch-clamp protocols and the *I_K_* curves are shown in **Figure 1A**. *I_K_* values for the control, MI-7d, MI-14d, fluvastatin-7d, and fluvastatin-14d groups were recorded separately. The current–voltage (*I*-*V*) curves were plotted as peak current density (defined as peak current amplitude/membrane capacitance of neurons) versus different membrane potentials (**Figure 1B**). The peak current densities of these five groups were 79.93 ± 17.35 pA/pF, 133.71 ± 23.70 pA/pF, 132.11 ± 27.41 pA/pF, 106.24 ± 11.93 pA/pF, and 99.25 ± 21.11 pA/pF (*n* = 10, *P* < 0.05), respectively. The peak current densities of the MI-7d and MI-14d groups were significantly higher than that of the control group (*n* = 10, *P* < 0.05). By contrast, the peak current densities of the fluvastatin-7d and fluvastatin-14d groups were significantly reduced compared to that of the MI groups (*n* = 10, *P* < 0.05).

### Effects of MI and Fluvastatin on Activation Kinetics of *I_K_*

Neurons were held at a holding potential of -40 mV and stimulated by a series of 160 ms step pulses from -40 to +50 mV (10 mV increment for each step), following a hyperpolarizing prepulse of -110 mV for 200 ms (**Figure 1C**). *I_K_* values for the control, MI-7d, MI-14d, fluvastatin-7d and fluvastatin-14d groups were recorded using the same stimulation procedures. The peak amplitudes of *I_K_* were converted into conductances using the equation *G* = *I*/(*V* -*V*_rev_), where *V* represents the membrane potential, and *V*_rev_ represents the reversal potential. The activation curves were fitted by conductance versus different membrane potentials to the Boltzmann equation *G*/*G*_max_ = 1/{1 + exp[(*V*_1/2_ -*V*)/*k*]}, where *G*_max_ is the maximum conductance, *V*_1/2_ is the membrane potential at half-activation, and *k* is the slope factor. The activation curves for *I_K_* of the five groups and the patch-clamp protocols are shown in **Figure 1C**. The values of *V*_1/2_ and *k* are listed in **Table 1**. The ANOVA showed that the five activation curves had no significant statistical difference (*n* = 10, *P* > 0.05). Fluvastatin and MI did not alter the activation characteristics of *I_K_*.

**Table 1:** Parameters of delayed rectifier potassium channel and sodium channel in five groups.

		Control group	MI-7d	MI-14d	Fluvastatin-7d	Fluvastatin-14d
			group	group	group	group
Delayed rectifier potassium channel	Potential at half-activation (mV)	4.40 ± 0.39	4.40 ± 0.96	–1.59 ± 1.24	–0.92 ± 2.14	–1.54 ± 1.10
	Slope factor of activation curve	21.60 ± 0.58	25.64 ± 1.65	27.20 ± 1.93	26.55 ± 4.42	26.50 ± 1.68
Sodium channel	Potential at half-activation (mV)	–40.69 ± 0.22	–48.26 ± 0.24	–48.31 ± 2.57	–43.53 ± 0.28	–42.80 ± 0.27
	Slope factor of activation curve	2.16 ± 0.56	3.09 ± 0.27	1.28 ± 1.93	2.66 ± 0.17	3.74 ± 0.23
	Potential at half-inactivation (mV)	–67.42 ± 0.38	–71.68 ± 0.45	–66.18 ± 0.43	–67.25 ± 0.42	–69.54 ± 0.48
	Slope factor of inactivation curve	7.73 ± 0.35	9.11 ± 0.41	8.65 ± 0.40	8.37 ± 0.38	9.82 ± 0.45
	Time constant of channel recovery (ms)	8.17 ± 0.62	3.75 ± 0.38	3.24 ± 0.39	4.12 ± 0.46	5.73 ± 0.49


*Values are presented as mean ± SD*.

### Effects of MI and Fluvastatin on I - V Curves of *I_Na_*

**FIGURE 2:**
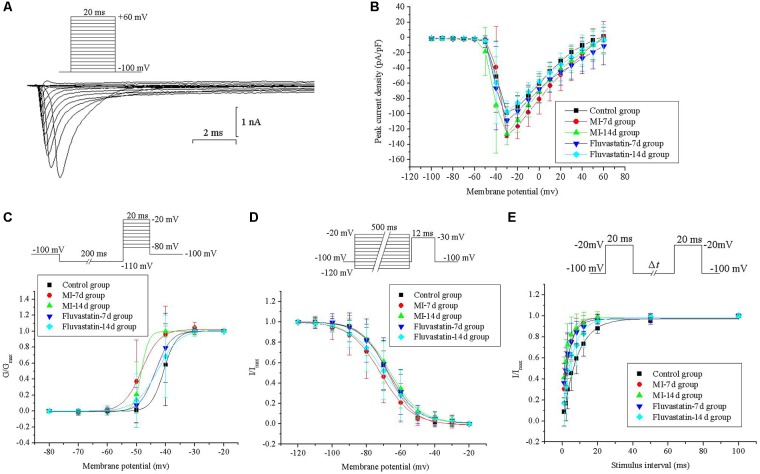
Effects of MI and fluvastatin on *I_Na_* of SCG neurons. **(A)** Typical examples of I_Na_ curves. **(B)** Effects of MI and fluvastatin on I–V curves of SCG neurons *I_Na_*. **(C)** Effects of MI and fluvastatin on activation process of *I_Na_*. **(D)** Effects of MI and fluvastatin on inactivation process of *I_Na_*. **(E)** Effects of MI and fluvastatin on recovery curves after inactivation of *I_Na_*. Each point represents mean ± SD.

Neurons were held at a holding potential of -100 mV and stimulated by a series of depolarizing steps from -100 to +60 mV (10 mV increment for each step, 20 ms pulse width). The fast activation and inactivation components of *I_Na_* were obtained. The patch-clamp protocols and the *I_Na_* curves are shown in **Figure 2A**. *I_Na_* values for the control, MI-7d, MI-14d, fluvastatin-7d and fluvastatin-14d groups were recorded separately. The *I* -*V* curves were plotted by adopting the above method and are shown in **Figure 2B**. The peak current densities were -98.65 ± 7.81 pA/pF, -127.98 ± 12.71 pA/pF, -126.32 ± 14.33 pA/pF, -108.74 ± 22.40 pA/pF, and -97.51 ± 14.55 pA/pF (*n* = 10, *P* < 0.05), respectively. The peak current densities of the MI-7d and MI-14d groups were significantly increased compared with that of the control group. The *I* -*V* curves of the fluvastatin-7d and fluvastatin-14d groups were not significantly different from that of the control group (*n* = 10, *P* > 0.05). Thus, fluvastatin reversed the changes of the *I* -*V* curves of *I_Na_* induced by MI.

### Effects of MI and Fluvastatin on Activation and Inactivation Kinetics of *I_Na_*

Neurons were held at a holding potential of -100 mV and stimulated by a series of step pulses from -80 to -20 mV (10 mV increment for each step, 20 ms pulse width), following a hyperpolarizing prepulse of -110 mV for 200 ms (**Figure 2C**). *I_Na_* values for the control, MI-7d, MI-14d, fluvastatin-7d and fluvastatin-14d groups were recorded separately. The activation curves for the five groups were plotted by following the above method and are shown in **Figure 2C**. The values of *V*_1/2_ and *k* in the five groups are listed in **Table 1**. The activation curves of the MI-7d and MI-14d groups were shifted toward the negative potential compared with that of the control group (*n* = 10, *P* < 0.05). The activation curves of the fluvastatin-7d and fluvastatin-14d groups were close to that of the control group (*n* = 10, *P* > 0.05). Thus, fluvastatin reversed the activation characteristics of *I_Na_* induced by MI.

Neurons were held at a holding potential of -100 mV and stimulated by a series of 12 ms test pulse of -30 mV, following a prepulse from -120 to -20 mV for 500 ms (Figure 1D). *I_Na_* values for the control, MI-7d, MI-14d, fluvastatin-7d and fluvastatin-14d groups were recorded using the same stimulation procedures. The peak amplitudes of *I_Na_* were normalized. The inactivation curves were fitted by *I*/*I*_max_ versus different membrane potentials to Boltzmann equation *I*/*I*_max_ = 1/{1 + exp[(*V* -*V*_1/2_)/*k*]}, where *V*_1/2_ is the membrane potential at half-inactivation, and *k* is the slope factor. The inactivation curves for *I_Na_* of the five groups and the patch-clamp protocols are shown in **Figure 2D**. The values of *V*_1/2_ and *k* in the five groups are presented in **Table 1**. The ANOVA of the five inactivation curves showed no significant statistical difference (*n* = 10, *P* > 0.05) between these groups. Thus, fluvastatin treatment and MI did not change the inactivation characteristics of *I_Na_*.

Neurons were held at a holding potential of -100 mV and stimulated by a series of double pulses (breadth: 20 ms, amplitude: -20 mV, intervals of double pulses (Δ*t*): 1, 2, 3, 5, 8, 12, 20, 50, and 100 ms) (**Figure 2E**). The control, MI-7d, MI-14d, fluvastatin-7d and fluvastatin-14d groups were stimulated using the same protocol, and *I_Na_* values were recorded. The peak amplitudes of *I_Na_* were normalized. The recovery curves after the inactivation of *I_Na_* were fitted by *I*/*I*_max_ versus different membrane potentials to the monoexponential equation *I*/*I*_max_ = 1-exp(-*t*/*τ*), where *t* is the recovery time, and *τ* is the time constant of channel recovery. The recovery curves after the inactivation of *I_Na_* of the five groups and the patch-clamp protocols are shown in **Figure 2E**. The values of *τ* are given in **Table 1**. The recovery curves after the inactivation of the MI-7d, MI-14d and fluvastatin-7d groups shifted toward the negative potential compared with the control group (*n* = 10, *P* < 0.05), and the recovery curves after the inactivation of the fluvastatin-14d group were similar to control levels (*n* = 10, *P* > 0.05). Therefore, the effects of fluvastatin on recovery curves after the inactivation of *I_Na_* may be correlated with the duration of MI injury.

### Effects of MI and Fluvastatin on RNA Expression of Channel Protein in SCG

**FIGURE 3:**
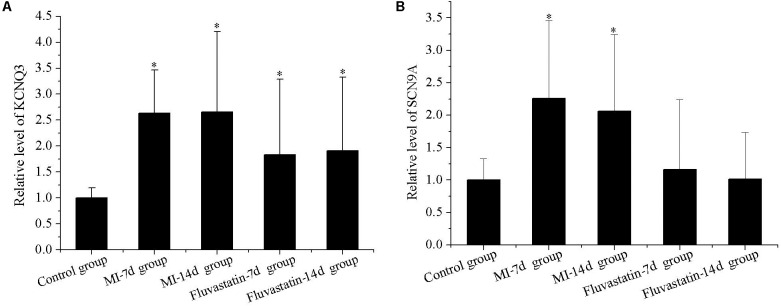
Effects of MI and fluvastatin on mRNA expression of channel protein in SCG neurons. **(A)** Effects of MI and fluvastatin on KCNQ3 expression. **(B)** Effects of MI and fluvastatin on SCN9A expression. Each point represents mean ± SD. ^∗^*P* < 0.05 compared with control group.

KCNQ3 and SCN9A expressions in the control, MI-7d, MI-14d, fluvastatin-7d and fluvastatin-14d groups are shown in **Figure 3**. The expressions of KCNQ3 and SCN9A in the MI-7d and MI-14d groups were significantly higher than that in the control group (*n* = 5, *P* < 0.05). The expressions of SCN9A in the fluvastatin-7d and fluvastatin-14d groups were not significantly different compared with the control group (*n* = 5, *P* > 0.05). Fluvastatin partially reversed the RNA expressions of the channel protein changes induced by MI.

### Effects of MI and Fluvastatin on Intracellular Calcium Concentration of SCG Neurons

**FIGURE 4:**
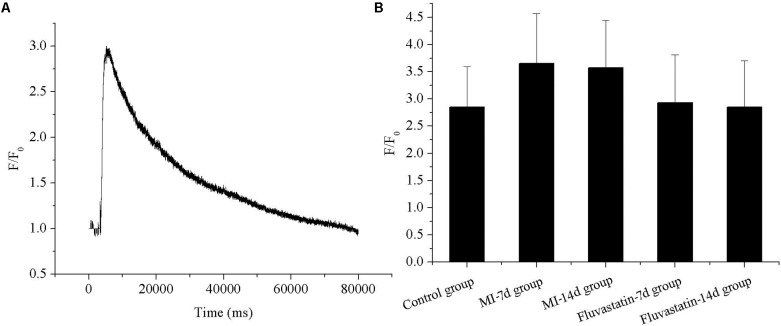
Effects of MI and fluvastatin on intracellular calcium concentration in SCG neurons. **(A)** Representative recordings of calcium concentration. **(B)** The histogram showed the average peak value of intracellular calcium concentration (peak fluorescence level F/resting fluorescence level F0) in five groups. Each point represents mean ± SD.

The representative fluorescence curve reflecting calcium concentration before and after adding 60 mM KCl are shown in **Figure 4A**. The calcium concentration (F/F_0_) in the control, MI-7d, MI-14d, fluvastatin-7d and fluvastatin-14d groups were 2.85 ± 0.74, 3.66 ± 0.92, 3.57 ± 0.88, 2.93 ± 0.88, and 2.85 ± 0.85, respectively (*n* = 10, *P* > 0.05). The respective histograms are shown in **Figure 4B**. The calcium concentration in the MI-7d and MI-14d groups were increased slightly; however, there were no significant differences in the five groups (*n* = 10, *P* > 0.05).

### Effects of MI and Fluvastatin on AP of SCG

**FIGURE 5:**
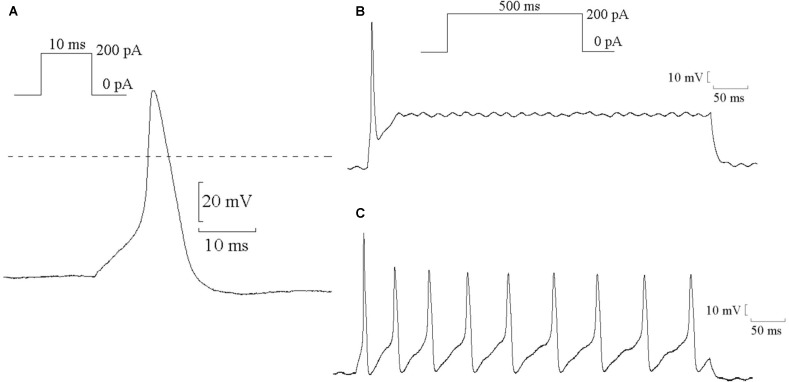
Typical examples of AP in SCG neurons. **(A)** Typical examples of single AP of SCG neurons were elicited by positive current 200 pA for 10 ms. **(B)** Typical examples of single AP were elicited by positive current 200 pA for 500 ms. **(C)** Typical examples of continuous AP were elicited by positive current 200 pA for 500 ms.

The single AP of SCG neurons were elicited using a positive current of 200 pA for 10 ms in the current-clamp mode. Representative single APs are shown in **Figure 5A**. The values of resting potential (RP), AP amplitude (phase 0), and APD_90_ (repolarization at 90% repolarization) of the control, MI-7d, MI-14d, fluvastatin-7d and fluvastatin-14d groups are presented in **Table 2**. Compared with the control group, the AP amplitude in the MI-7d and MI-14d groups were significantly higher (*n* = 10, *P* < 0.05), and APD_90_ levels were significantly shorter (*n* = 10, *P* < 0.05). Fluvastatin treatment reversed AP amplitude and APD_90_ levels (*n* = 10, *P* > 0.05).

**Table 2:** The parameters of AP in five groups.

	Control group	MI-7d group	MI-14d group	Fluvastatin-7d group	Fluvastatin-14d group
Resting potential (mV)	–60.39 ± 2.48	–57.86 ± 5.31	–59.55 ± 9.37	–58.73 ± 8.13	–60.38 ± 10.29
Amplitude of AP (mV)	95.98 ± 11.38	100.93 ± 8.79*	102.93 ± 6.80*	97.14 ± 13.12	98.76 ± 12.86#
APD_90_ (ms)	9.45 ± 2.92	7.47 ± 0.70*	7.46 ± 1.04*	8.94 ± 1.18	8.79 ± 1.66


*Values are presented as mean ± SD, ^∗^*P* < 0.05 compared with control group. ^#^*P* < 0.05 compared with MI 7 days group.*

Continuous AP was elicited by a current of 200 pA for 500 ms that can induce single AP (**Figure 5B**) or continuous AP (**Figure 5C**). The probability of the induced continuous AP (the number of neurons that induced continuous AP/the total number of neurons that recorded correct AP) of the control, MI-7d, MI-14d, fluvastatin-7d and fluvastatin-14d groups were 38.24, 86.36, 68.00, 55.00, and 51.35%, respectively. Therefore, fluvastatin prevented the change of characteristics of AP induced by MI and exerted protective effects on the electrical activity of SCG neurons.

## Discussion

### Major Findings

This is the first study that assessed the SCG neuron ion channel characteristics in different time points following ischemia and the effects of fluvastatin upon these. The major findings are as follows. (1) Delayed rectifier potassium and sodium channel peak current densities in SCG neurons were significantly increased in the MI group, as reflected in the higher KCNQ3 and SCN9A mRNA expression. (2) The mean AP amplitude of the neurons in the MI group was higher and APD_90_ was shorter. (3) The intracellular calcium concentration in SCG neurons was slightly increased during MI. (4) The ion channel characteristic, the mRNA expression of channel protein, and the AP characteristic of SCG neurons were significantly altered in different ischemic durations. (5) Fluvastatin treatment partly recovered these changes in the two MI groups.

#### AP Change in SCG Neurons of MI

The occurrence and development of different cardiovascular diseases are closely related to sympathetic system activation (Hernández-Ochoa et al., 2009; He et al., 2012; Kong et al., 2013; Murakami et al., 2015). Some nerve fibers of the SCG directly innervate the heart. Sympathetic ganglion blockade has been used for the treatment of heart disease and protection of the cardiac function (Na et al., 2014). Single neurons in the SCG are important mediators of the electrical function of the heart. Compared with the sympathetic nerves located within the heart, SCG tissues were relatively larger and obtaining single neurons for experimentation was easier.

Our experimental results showed that MI injury significantly changes the characteristics of SCG neurons. Previous work has shown that MI causes an increased activity of sympathetic nerve and norepinephrine (NE) released from the sympathetic nerve endings in the myocardium (Robador et al., 2012; Wu et al., 2012; Hall et al., 2016; Fukui et al., 2017). The probability of inducing continuous AP in SCG neurons was increased in MI groups.

#### Relationship Between AP Changes and Ion Channel Remolding in MI

The AP of neurons was made up of a variety of ion currents and was directly affected by the ion channel changes (Dai et al., 2016). Our previous experiments have revealed that the inward sodium channel and the outward delayed rectifier potassium channel are the two main ion channels found on the neuronal plasma membrane, and that changes in their properties or expression levels would exert significant alterations in the AP characteristics. The major component of the sodium channel of SCG neurons is the SCN9A (Nav1.7) subunit (Ahn et al., 2011) that plays important physiological roles in AP initiation and propagation (Barker et al., 2017), and the resulting sodium current governs the AP amplitude. Our results show that MI injury causes the peak current density of the *I_Na_* to increase, leading to the increase in the amplitude of the AP. The major component of the delayed rectifier potassium channel current is the KCNQ2/KCNQ3 (Kv7.2/Kv7.3) subunits (Shapiro et al., 2000; Matsumoto et al., 2010), and is one of the major currents in the AP repolarization course (Zhang et al., 2018) and directly affects the AP repolarization time (Yeo et al., 2017). Our results show that MI leads to an increase of the peak current density of the delayed rectifier potassium channel current. Sodium channel and delayed rectifier potassium channel protein mRNA expressions also increase compared to the control group. Together, these findings relate the higher AP amplitude and the shorter APD_90_ observed after MI to the increasing sympathetic activity and the subsequent NE release.

The activation of sympathetic nerves is altered with the different ischemic durations. Previous work has shown that the incubation of cardiac synaptosomes in hypoxic conditions for 30 min causes a ≥50% increase in NE release (Robador et al., 2012). One week after MI, the sympathetic remodeling of the heart significantly increases (Wu et al., 2012). Our results show that ischemia at 7 and 14 days increases the neuronal activity, including the ion channel function and expression and the consequent changes in AP waveforms, without significant differences between the two time points.

#### Intracellular Calcium Concentration in SCG Neurons

Increases in intracellular calcium concentrations trigger different physiological processes, such as muscle contraction, transmitter release, hormone secretion, and so on. The neural electrical activity is increased by escalating changes of intracellular calcium concentration (Wang et al., 2015). The voltage-gated calcium channels are the main regulators of the intracellular Ca^2+^ level. The main calcium channel is the N-type calcium channel [CACNA1B (Cav2.2)] in the peripheral nervous system that includes SCG neurons (Zhou et al., 2017). Previous experiments have found that the N-type calcium channel current of sympathetic ganglion neurons innervating the heart is higher in spontaneously hypertensive rats than in normotensive rats (Larsen et al., 2016). Our results show that the intracellular calcium concentration in SCG neurons is slightly increased during MI. However, because no significant changes in the intracellular calcium concentrations in SCG neurons were detected between the different groups, N-type calcium channels were not studied further in the present study. These results suggest that electrical remodeling in SCG neurons is mainly because of sodium channels and delayed rectifier potassium channels, rather than calcium currents.

#### Roles of Fluvastatin Treatment in MI

Statins exert cardiovascular protective effects independently of their cholesterol-lowering effects (Ambrosi, 2013; Schwartz et al., 2017). Fluvastatin is an example of this class of medications (Zhou et al., 2008; Schouten et al., 2009). Fluvastatin ameliorates cardiac sympathetic neural dysfunction in diabetic rats (Matsuki et al., 2010). In addition, clinical and experimental studies have suggested that statins decrease sympathetic activity and effectively ameliorate cardiac sympathetic nerve remodeling (Kim et al., 2009; Lewandowski et al., 2014; Yang et al., 2016), but its mechanism of direct protection actions on sympathetic nerve has not been fully characterized. In our experiments, the characteristics of the SCG including the electrical properties and mRNA expression of ion channels, as well as AP characteristics were significantly altered at different time points after MI. Fluvastatin pretreatment reversed these changes in SCG neurons caused by MI injury. Fluvastatin can reduce the electrical activity of SCG neurons, consistent with the results in clinical and experimental studies that statins decrease sympathetic activity and effectively ameliorate cardiac sympathetic nerve remodeling (Kim et al., 2009; Lewandowski et al., 2014; Yang et al., 2016).

#### Isoproterenol-Induced MI Model

Surgical ligation and drug injection are two commonly used methods of MI models. In our experiments, single SCG neurons were obtained for patch-clamp experiments from young rabbits rather than adult rabbits. Ligation led to a highly mortality rate in young rabbits due to bleeding and great trauma caused by thoracotomy. The injection method can significantly reduce the mortality rate. Furthermore, by using the injection method, we can simulate the vasoconstriction process of MI by increasing the myocardial contraction force, shrinking the coronary artery, and increasing the myocardial oxygen consumption. These pathological processes are similar to human ischemia or anoxic MI (Zhou et al., 2008). Therefore, we chose the ISO-induced MI method.

#### Limitations

Several limitations of this study should be noted. Firstly, mRNA expression of SCN9A and KCNQ3, rather than KCNQ2 expression in SCG neurons were analyzed in the present study as the rabbit gene sequence was not found in the NCBI website. Secondly, the extent to which N-type calcium channels [CACNA1B (Cav2.2)] play a role in SCG neuron activity is unclear, though our study found no significant difference in calcium handling whether following MI alone or with fluvastatin treatment.

## Conclusion

In summary, MI increased the SCG neuron activity. Fluvastatin treatment before MI partly reversed the characteristics of SCG neurons of rabbits. The present study suggested that the protective mechanism of statins could be achieved through its influence on sympathetic ganglion neurons dominating the heart. The statin’s protection role of reducing sympathetic nerve electricity activities of SCG neurons might be a new target of cardiovascular treatment.

## Author Contributions

LC and GL conceived the work, designed the experiments, collected and analyzed data, and drafted the manuscript. XW and TL recorded and analyzed the experimental data. HF and GT interpreted the data and critically revised the manuscript.

## Conflict of Interest Statement

The authors declare that the research was conducted in the absence of any commercial or financial relationships that could be construed as a potential conflict of interest. The reviewer JB and handling editor declared their shared affiliation at the time of the review.

## Abbreviations

SCG: superior cervical ganglion
MI: myocardial ischemia
APD90: the time of the repolarization up to 90% of AP
AP: action potential
SD: standard deviation
*I_Na_*: sodium channel current
*I_K_*: delayed rectifier potassium channel current
*I*–*V*: current–voltage
ISO: isoproterenol
